# Efficient Data Collection in Widely Distributed Wireless Sensor Networks with Time Window and Precedence Constraints

**DOI:** 10.3390/s17020421

**Published:** 2017-02-22

**Authors:** Peng Liu, Tingting Fu, Jia Xu, Yue Ding

**Affiliations:** Key Laboratory of Complex Systems Modeling and Simulation, School of Computer Science, Hangzhou Dianzi University, Hangzhou 310018, China; perryliu@hdu.edu.cn (P.L.); knowyouabc@163.com (J.X.); daisy_ding1992@outlook.com (Y.D.)

**Keywords:** data collection, widely distributed sensor networks, Unmanned Ground Vehicle, mobile sink, precedence constraints, trajectory planning

## Abstract

In addition to the traditional densely deployed cases, widely distributed wireless sensor networks (WDWSNs) have begun to emerge. In these networks, sensors are far away from each other and have no network connections. In this paper, a special application of data collection for WDWSNs is considered where each sensor (Unmanned Ground Vehicle, UGV) moves in a hazardous and complex terrain with many obstacles. They have their own work cycles and can be accessed only at a few locations. A mobile sink cruises on the ground to collect data gathered from these UGVs. Considerable delay is inevitable if the UGV and the mobile sink miss the meeting window or wait idly at the meeting spot. The unique challenge here is that, for each cycle of an UGV, there is only a limited time window for it to appear in front of the mobile sink. Therefore, we propose scheduling the path of a single mobile sink, targeted at visiting a maximum number of UGVs in a timely manner with the shortest path, according to the timing constraints bound by the cycles of UGVs. We then propose a bipartite matching based algorithm to reduce the number of mobile sinks. Simulation results show that the proposed algorithm can achieve performance close to the theoretical maximum determined by the duty cycle instance.

## 1. Introduction

In recent years, Wireless Sensor Networks have tended to be deployed in a wide area such as forests [1], cities [2], and underwater [3]. With the development of unmanned vehicles, sensors now can be mounted on them to extend their exploring scope. Normally, these vehicles are far from each other and require data collectors to hand in their data. For example, Autonomous Underwater Vehicles (AUVs) have been deployed in the deep sea [4,5] to do data collection and event detection. They will appear frequently for data reporting to avoid underwater acoustic transmission, which suffers from very significant signal attenuation [6]. When they are underwater, their exploration work is often with a fixed schedule since the trajectory and working spots are predetermined. After these AUVs surface, a moving mobile sink will come to collect the data via wireless communications. UGVs are also favorable for sensor network applications [7]. The mobile sink cruises in the area and visits each unmanned vehicle in a timely manner and uploads collected data via a satellite communication. In these scenarios, a unique problem is how to maximize the data collected via the trajectory planning of the mobile sink.

To deploy a stationary data collector at the spot of of each UGV appearance is costly and not feasible in some cases, e.g., ocean bottom surveillance scenarios, since it is impossible to anchor the collector in the deep sea without it drifting away. Therefore, using a mobile sink is feasible and economical. The key challenge is that the mobile sink must arrive at the spot at a certain time to meet the UGV according to its work cycle. Arriving before the UGV appears or after it goes back will cause the data collecting procedure to fail. Therefore, the constraints are not only the shorter path but also the timing opportunities. In traditional scenarios, the problem can be fully or partly converted to the Traveling Salesman Problem since the node is always available. However, in our case, the data generators will only allow a short meeting time during a periodical cycle so that the visiting sequence and distance between two nodes will have a great impact on the global performance.

**Figure 1:**
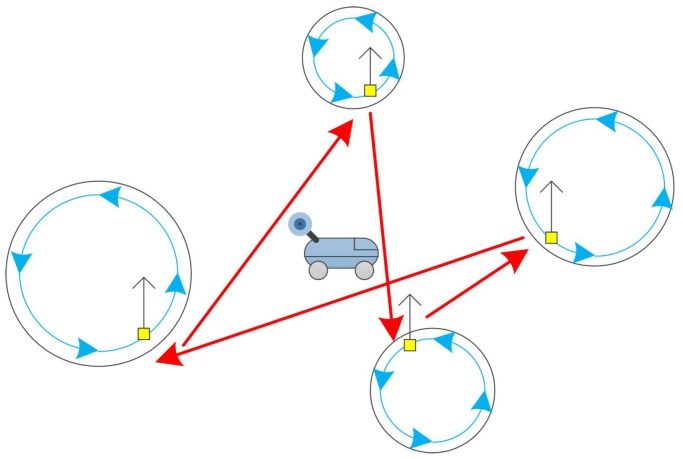
Sensor network that monitors a certain area with multiple events.

There is previous work studying the trajectory planning of mobile nodes in fragmented wireless sensor networks [8,9,10,11]. In our paper, we consider a common scenario in which UGVs are working in the obstacle area to collect data. They will appear at some locations and hand data to the mobile sink cycle by cycle. UGVs contact the mobile sink in a wireless communication and the latter one uploads the data to the satellite. Figure 1 shows the proposed scenario. The target area is divided into several sub-parts since it is impossible for a single UGV to explore the entire area. Therefore, each UGV will work in its own sub-part independently where the acreage, the environment, and the tasks may be quite different. In other words, each cycle of the UGVs is different. Even for the same UGV, the cycle time is not identical due to unexpected events and condition of the path. Since this will introduce extra delay during the process of reappearing or resurfacing [3], a UGV will appear only for a short time in each cycle. Without loss of generality, in this paper, we assume that each UGV will only appear for one time during a cycle as shown in Figure 1. Furthermore, the UGV will not stay long at the appearing spot. In other words, the size of the time window is negligible compared with the distance between appearance spots.

In this paper, we consider the trajectory planning of the mobile sink aiming at maximizing the collected data during a given time period, which, in turn, minimizes the average data reporting delay. After that, if there exists more than one path that passes the same amount of UGVs, we will further identify the shortest path among them. The contribution is quite unique compared with previous mobile sink based problems because there is only a tiny time window for the data collector to meet with each UGV, for which the exact appearance time is also inaccurate. If all the UGVs have the same cycle, the problem can be recognized as a Travelling Salesman Problem (TSP). If each UGV has a different cycle, it can be converted into a layered TSP problem. However, the speed of the mobile sink depends on how these sub-cycles are formed. These approaches focus on the shortest path while ignoring the precedence relationship of the UGVs. Deadline driven methods deal with the situation that each UGV must be visited according to some kind of sequence (energy depletion, buffer overflow, etc.). However, meeting the current deadline of a UGV may result in missing that of another two in the future since the sequence is also affected by the distance between two UGVs. There is also related work on TSP with a time window [12,13,14]. However, although our work looks similar to these papers, it is distinguished from these special cases of the TSP problem. First, the mobile sink is not required to return the start point. Second and most important, the network graph keeps changing along the time scale. The problem is not as hard as the TSP problem due to timing and precedence constraints. The search space is reduced to a limited area. Third, the object is to visit as many UGVs as possible in a period. The k-hop based search algorithm and bipartite matching based minimal mobile sinks finding algorithm are the main originalities of our work.

Our contribution is four-fold:

1. We consider the trajectory planning problem with time window and precedence constraints which evolves throughout time.
2. We develop optimal algorithms to solve the trajectory planning of the mobile sink in the ideal case.
3. We consider the situation cycles are changing dynamically and develop a greedy algorithm to solve it.
4. We develop a method to calculate the optimal number of mobile sinks when the area is too large for a single mobile sink to serve.

The rest of the paper is organized as follows: Section 2 discusses the issues in the existing work and our research incentive. Section 3 presents the problem formulation. Then, we give details of the trajectory planning implementation in Section 4. The algorithm for calculating an optimal number of mobile sinks is presented in Section 5. The effectiveness and improvement of our method are shown with the experimental results in Section 6. Section 7 concludes this paper.

## 2. System Background and Related Work

In this section, we summarize research work related to trajectory planning of mobile nodes. Using mobile sinks to collect data has been a hot topic in Wireless Sensor Networks (WSNs). Ref. [15] tries to determine the constant velocity of the mobile sink with a fixed-mobility pattern so that data collection throughput can be maximized. Ref. [16] addresses the problem of finding an optimal path for the mobile sink to collect packets from each sensor such that the total energy use is minimized and is subject to the length constraint. Ref. [17] studies the problem of controlling sink mobility in event-driven applications to achieve a maximum network lifetime by a convex optimization model inspired by the support vector regression technique. Ref. [18] considers a delivery latency minimization problem (DLMP) in a randomly deployed WSN and solves it using the traversed anchor points on the border of the communication range of sensor nodes to shorten the travel path, along with the movement of the mobile sink. All of these works focus on the shortest path under different conditions. In [19], a framework is built for investigating the joint sink mobility and routing problems by constraining the sink to a finite number of locations. They formally prove the NP-hardness of the problem. They also investigate the induced subproblems. In particular, they develop an efficient primal-dual algorithm to solve the subproblem involving a single sink and then generalize the algorithm to approximate the original problem involving multiple sinks. Some work [20] considers the constrained path of the mobile sink while our work considers the constrained timing allowance. Guangqian Xie [21] considers the obstacle constraints in the mobile sink path scheduling. However, it is still a case of location constraints. To harvest the full potential of nodeąŕs mobility, Omar Aldabbas [22] presents an algorithm to allow sensor nodes to select the mobile sink that offers the best service for the longest period. In the approach, the data still needs go through a few relays to reach the mobile sink.

Some research has been done about the TSP problem with a time window that looks very similar to our work. In [12], the authors propose the time-constrained TSP problem where each city must be visited during a time window. They use a parallel 2-exchange local search algorithm and extend it to multiple time windows. The algorithm can run on a multi-processor computer and achieve the time complexity of $O(\log^{2}n)$. As well as the time window, Fagerholt et al. [13] also consider the precedence constraints which require ports being visited sequentially in a real ship scheduling scenario. They proposed a forward dynamic programming algorithm and proved that it works efficiently. In [14], a multi-traveling salesman problem with a time window and precedence constraints are further considered. The author claims that the problem is NP-hard and proposes the Linear Programming relaxation of the set partitioning formulation by column generation. Then, a special branch-and-bound scheme is employed to solve the integer set partition formulation. The algorithm is capable of optimally solving 30 customer problems. Basagni et al. [23] consider the decaying of the data value in an underwater sensor network. They try to find the trajectory and floating spots that can maximize the value of the data collection. However, the problem proposed in our paper is quite different from these related works. First, we do not require the trajectory of the mobile sink to be a loop. Second, the graph keeps changing along the time scale. Third, the object is not to find the shortest path but to visit as many UGVs as possible in a period.

To find the minimal number of vehicles needed to serve the set of locations in a *multiple traveling salesman problem with time windows* (m-TSPTW) [24], two precedence graphs are introduced as well as algorithms to find a lower and an upper bound. The start-time precedence graph and end-time precedence graph represent the left and right bound of the time window respectively. However, the two bounds are handled separately so that many available paths are omitted. Although the maximum bipartite matching is mentioned for finding the minimal number of vehicles, the paper does not give any detail on this.

All of the above work fails to solve the path planning along the time scale. In addition, they cannot deal with the situation of an inconsistent window. In [25], the changing of time windows and precedence constraints due to continuous intelligence updates is considered. However, the authors are not aiming at tackling the problem directly, but by solving the related TSP and applying the solutions to the change restrictions until one of them satisfies the conditions optimally. The so called *k*th best solution strategy will have a high overhead when the restriction changes quickly.

## 3. System Model and Problem Formulation

In this section, we will formulate the system model and explain the unique changeless of the proposed problem.

### 3.1. UGV Appearance Cycles

To visit UGVs successfully, the mobile sink needs to know the exact time and location when the UGVs will appear. According to [3], once the traveling cycle of the search space is known, the UGV reappearing frequency *k* can be determined to minimize the average data delay. However, the reappearing frequency *k* is only a theoretical value, since many facts will affect the movement of the UGVs, such as ocean current and terrain of the seabed. Each UGV will try to follow their schedule but the actual reappearing time varies. In this paper, we model the duration between appearances of UGVs using Gaussian distribution [23] (i.e., *X*∼$P(\mu ,\sigma^{2})$). Each UGV is independent from others. Since it is not possible to guarantee a 100% chance of encounter, we set up a threshold *δ* (e.g., δ=0.9, which stands for the confidence possibility of 90%). To meet with the target UGV *ν* with confidence possibility of *δ*, the mobile sink must arrive at the spot between $t_{s}^{\nu }$ and $t_{e}^{\nu }$. Equation (1) shows how to calculate $t_{s}^{\nu }$ and $t_{e}^{\nu }$, where $t_{s}^{\nu }$ and $t_{e}^{\nu }$ stand for the left bound and right bound, respectively, of the estimated reappearance duration of *v*

(1) $$\sum_{x=t_{s}^{\nu }}^{t_{e}^{\nu }}P\left(x,\mu ,\sigma^{2}\right)\geq \delta \;|\;where\;\mu -t_{s}^{\nu }=t_{e}^{\nu }-\mu .$$

To make it simple, we assume that the time window of the UGV appearing is very short compared with the time elapsed during traveling since the UGV needs to work and cannot spend much time waiting. Therefore, the appearance time of an UGV can be regarded as a point along the time scale. In the static scenario, a time window with lower and upper bounds can be solved by adding virtual points. However, it is impossible for the scenario proposed in this paper since the number of points is very large.

### 3.2. Mobile Sink Trajectory Planing

**Figure 2:**
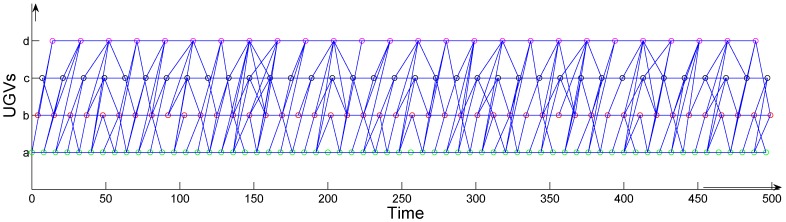
All possible edges in the precedence graph.

To plan the trajectory of the mobile sink, we create a precedence graph where the *x*-axis is time and the *y*-axis is the individual UGVs. Suppose that there are a total of four UGVs represented by *a*, *b*, *c*, and *d*. Step 1, as seen in the Figure 2, each *y* represents a UGV, and, supposing the UGV appears at time *t*, we then plot a point at the cross. For the same *y*, we will get many isolated points along the time scale. If a deadline is given, we could count the total number of points (noted as *n*) from all of the UGVs. As shown in Figure 2, when the *x* reaches 500, the total number of points is 172 (*n* = 172). In Step 2, we add edges between these points. Suppose that there are two points *a* and *b*, and $t_{a}$ represents the time that *a* appears. $d_{ab}$ is the time that the mobile sink takes to move from *a* to *b*. If $t_{b}-t_{a}\geq d_{ab}$, then we draw an edge between *a* and *b*, and add the edge $e_{ab}$ into the adjacency matrix. To reduce the search space, for each point, it will have a number of edges that is no more than the number of the neighboring UGVs. In other words, a UGV will generate many points, and a point generated by another UGV will build an edge to the nearest feasible one among them.

A matrix *M* is derived from Figure 2. We sort all the points according to their appearance time along the time scale in the figure and number them from 1 to *n*. The row and column elements of the matrix are both these points where we have

(2) $$\begin{array}{c} M_{ij}=\left\{\begin{array}{cc} d_{ij}, & \mathrm{if}\;t_{j}-t_{i}\geq d_{ij},\\ -1, & \mathrm{otherwise}, \end{array}\right. \end{array}$$

(3) $$AdjecencyMatrix=\begin{array}{cc} & \begin{array}{ccc} \;0\; & \;1\; & \;2\; \end{array}\\ \begin{array}{c} 0\\ 1\\ 2 \end{array} & \left(\begin{array}{ccc} -1 & 1 & 1.5\\ -1 & -1 & 1\\ -1 & -1 & -1 \end{array}\right) \end{array}.$$

An example of the matrix is shown in Equation (3), which is related to a triangle graph where three points are located at the vertexes. If there is a non-negative value at the crossing of two points, it means that the mobile sink can move from one point to another without missing the data collection window. The value is the time needed to travel from one point to another. Any previous work based on TSP can not solve this problem, since the node is only accessible at a certain time, and each cycle is so different.

## 4. Optimizing Path Schedule of the Mobile Sink

In this section, we develop algorithms to optimize the path scheduling of the mobile sink so as to visit as many UGVs as possible.

### 4.1. The Ideal Case

In this section, we first try to find a solution to solve the path optimizing problem of the mobile sink when each UGV follows its monitoring cycle strictly. Therefore, we have all the information to guide the scheduling. As described above, we now have the spatial-temporal graph of the system. The goal is to find a trajectory of the mobile sink that could visit UGVs as many times as possible. The problem is unique and quite different from the traditional salesman problem, i.e., the TSP problem. A TSP problem is trying to find the shortest path that visits all the points from the starting point and finally returns to the same point. The graph is a precedence graph that can not be solved using traditional algorithms.

**Definition** **1.** A path that visits all points in the precedence graph is called a **complete path**.

**Definition** **2.** If b is to the right of a and there is an edge linking them, we say that b is a’s **neighbor**. In other words, b appears later than a in the graph.

**Definition** **3.** If b is the closest point to a among all neighbors of a in the time scale, we say that b is a’s **nearest neighbor**.

We have Theorem 1 that shows our problem is not equal to the traditional TSP problem since only one complete path exists, so it is not necessary to find the shortest path.

**Theorem** **1.** If paths passing all points in the given Graph do exist, they are the same path.

**Proof.**  Suppose there exists two different paths that pass all points, i.e., $P_{1}$ and $P_{2}$. $P_{1}$ has the point sequence of $\left(v_{0},v_{1},\ldots ,v_{i},\ldots ,v_{j},\ldots v_{n}\right)$. Then, there exists at least two points having different sequences in $P_{2}$, e.g., $v_{i}$ and $v_{j}$. However, if there is path between $v_{i}$ and $v_{j}$, then $t_{v_{j}}$ must be greater than $t_{v_{i}}$. Therefore, the path is not valid from $v_{j}$ to $v_{i}$, which means path $P_{2}$ is not a valid path. ☐

**Theorem** **2.** The necessary and sufficient condition under which we could find a complete path in a given graph is that each point has an edge to its nearest neighbor.

**Proof.**  Suppose there exists at least two points $v_{i}$ and its nearest neighbor $v_{j}$, and there is no direct edge between them. Therefore, the path connecting $v_{i}$ and $v_{j}$ must go through at least one middle point $v_{k}$. However, since $v_{j}$ is already the nearest neighbor of $v_{i}$, there is no point $v_{k}$ between them, i.e., $v_{k}$ does not exist at all. Thus, there is no such path that can visit both $v_{i}$ and $v_{j}$. ☐

From Theorems 1 and 2, we propose the algorithm 1 to find the complete path in the given graph.
**Algorithm 1**
**Complete path** finding algorithm in the precedence graph**Require**: adjacency matrix M **Ensure**: a point sequence $P=\{v_{0},v_{1},\ldots ,v_{m}\}$ forming a path.
1:Set current point *q* to the leftmost point in the graph (starting from the element M[q,q] where q=0), P={q}2:For current point *q*, find its **nearest neighbor** *r* in the graph (the first element M[q,r] that has a non-negative value). If there are no more neighbors, the algorithm ends.3:Add point *r* into the *P*. Then, P=P∪{r}4:Set q=r as the current point, and GOTO step 2


The algorithm is very simple, according to Theorem 2. At each step, we only need to follow the edge to the nearest neighbor of the current point. Finally, all of the edges will form a path from the first point to the last. If the same pattern appears somewhere along the time scale, then it can be declared that an effective cycle has been found. Since there is only one complete path, it is also the shortest path.
**Algorithm 2**
**Optimal shortest path** finding algorithm in the precedence graph**Require:** adjacency matrix M **Ensure:** hop number $s_{i}$ of each point *i* and a point sequence $P=\{v_{0},v_{1},...,v_{m}\}$ forming a path.     **Initialize**
1:Set current point *q* to the rightmost point in the graph (starting from the element M[q,q] where q=n).**Mark**2:For current point *q*, find a point *r* with the largest hop number and shortest path length among all *q*’s neighbor, and set $s_{q}=s_{r}+1$, $l_{q}=l_{r}+d_{qr}$. If there is no neighbor, set $s_{q}=1$, $l_{q}=0$.3:If *q* is the leftmost point, GOTO step Finish. Otherwise, set the first point to the left of the current point *q* (i.e., q=q-1) as the current point, GOTO step Mark.**Finish**4:Start from the point(s) with the largest hop number and smallest path length, add it into *P*, and recursively add its neighbor with the hop number decreasing by one. When there is more than one point having the same largest hop number, the algorithm records all the paths.


However, in many cases, it is impossible to find a trajectory of the mobile sink to visit every UGV upon their appearance—e.g., when the interval of two UGVs reappearing is less than the traveling time between them, there is no doubt that one mobile sink cannot reach them both. In this case, the objective is to visit as many UGVs as possible so that more data can be collected in time. To find a path that visits the most number of UGVs, the easiest way is to exhaust all possibilities. The time complexity, which is $O(n\times 2^{n})$, increases quickly as the number of UGVs increases. In this paper, we propose a simple algorithm (Algorithm 2) that achieves the same goal, taking advantage of the precedence graph.

**Definition** **4.** The term **hop number** of point q stands for the optimal number of points that can be visited until the rightmost point when starting from q.

**Definition** **5.** The term **path length** of point q (denoted as $l_{q}$) stands for the sum of all edges from q to the rightmost point with hop number of 1.

The algorithm starts from the farthest right point on the time scale, and then scans and marks from the right to the left one by one. At each point, it tries to connect to the neighbor with the longest path. Scanning along the time scale ensures that no point is ignored and is in accordance with the precedence requirement. The time complexity is O(l×n)→O(n), in which *n* is the total number of points in the precedence graph, and *l* is the number of UGVs (l≪n). There may exist more than one optimal path. In the TSP problem, it is NP-Complete to find the shortest path. However, since there are time window and precedence constraints, and a point will not have more than *l* edges, the combination in our case is very limited. In the Mark phase of the algorithm 2, it is easy to choose the neighbor of the shortest path by recording the neighbor with the shortest path.

**Theorem** **3.** Algorithm 2 can obtain the optimal path that contains the largest number of points (UGVs).

**Proof.**  We use mathematical induction. Supposing that there is only one point, the algorithm obviously works. Then, suppose that the algorithm finds the optimal path(s) starting from a set of points *Q* when there are *n* points. If we insert one more point *r* on top of it that increases the total number to n+1, without loss of generality, we can always add it to the left side of the other points since the algorithm starts from the rightmost and searches to the left. If there is an edge between *r* and any point in *Q*, according to the algorithm, *r* will be added to the optimal path. When there is no edge between *r* and any point in *Q*, no matter which path *r* will join, the hop number of it can not be greater than $s_{Q}$ since all other points have smaller hop numbers (at least one less) than $s_{Q}$. In that case, if $s_{r}$ equals $s_{Q}$, *r* will be added into the point set *Q*. Therefore, for n+1 points, the algorithms still works. ☐

### 4.2. The Practice Case

As we mentioned before, in practice, each UGV is not able to follow their schedule strictly due to the various facts. Most of the time, they will arrive slightly ahead or later than scheduled, while, in rare cases, there is a large deviation. Therefore, it is not possible to build a precedence graph since the future is not predetermined and predictable so that “backward” algorithms cannot work anymore. In this paper, according to the statistics, we model the interval between two appearances of a UGV as the Gaussian distribution. Only the expected time of the first appearance and the expected cycle time of each UAV are known to the mobile sink. All other additional information, such as the exact appearing time of a UGV in a certain cycle, can only be received when the mobile sink visits a UAV and meets it during its appearance. Therefore, if any proactive solutions still rely on the precedence graph without timely information updating, a time interval for the mobile sink arriving early and staying long enough at each UGV point must be preserved on the base of the worst case of the possibility. However, the consequence is a sharp degression in performance due to accumulated errors.

**Figure 3:**
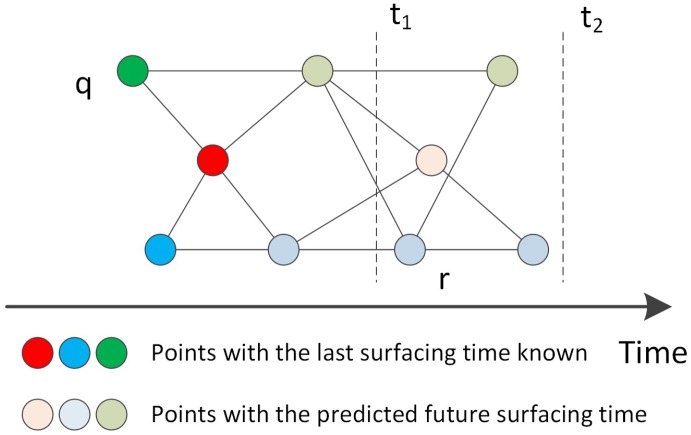
k-hop search for a better path (k = 3) (the coordinate of the point does not represent the physical location, and the length of an edge does not reflect the distance between two points).

In this paper, we propose a distributed greedy algorithm called **k-hop** (Algorithm 3) to solve the problem. *k* means the depth of the search algorithm. As shown in Figure 3, there are three UGVs, and nine points are generated along the time scale. Points with a darker color are the last appearance times known, while points with a lighter color are the predicted appearance times. The search starts from the point *q* and expands the path by adding points until the path reaches three edges. Many paths may exist, and the points at the end of those paths are called *q*’s k-hop neighbors. As seen in the Figure 3, where *k* = 3, these points in the area between the line $t_{1}$ and $t_{2}$ are all k-hop neighbors of *q*. In other words, the mobile sink needs to cross three edges (hops) to reach any of those points. Since it is not possible to fully predict the future, in order to visit as many points as possible, a local optimal can be achieved instead, where we need find the path passing the same number of points in the least amount of time. In Figure 3, when *k* = 3, there are two paths leading to the nearest 3-hop neighbor, point *r*. We can pick any one of them to start the next round search. The simplest way is giving *k* the value of “1”. Then, the algorithm will set the nearest neighbor in the time scale of the current point as the next point. Apparently, the greater the *k* is, the more possibilities we have in finding the optimal path. However, since the UGVs appear irregularly, the more hops we consider, the more errors as well as computational costs will be introduced. There must be a trade-off between the performance and the cost.

In the worst case, the complexity of the k-hop algorithm is $O(n^{k})$. Therefore, we must find the balance of the performance and the overhead of the *k*. In this paper, we propose the empirical value of *k* as

(4) $$k=\log_{{\bar{A}}_{out}}n,$$

where *n* is the total number of points in the precedence graph and ${\bar{A}}_{out}$ stands for the averaged out degree in the graph. The idea behind the equation is that if the averaged out degree is large, then the algorithm can search closer for a better path since multiple paths overlap at many common points. In a few steps, different paths may be connected to the same point. On the other hand, when ${\bar{A}}_{out}$ is small, the path is more independent so the algorithm needs to check more steps before it can make a decision on which one is better because, if the chosen path is not good, the mobile sink has to stay in this path for some steps before it can choose another path. The shortest path can also be achieved based on our algorithms since, in the matrix *M*, the distance between two points is recorded. It is not difficult to calculate the shortest path.
**Algorithm 3**
**k-hop** path finding algorithm in the precedence graph**Require:** adjacency matrix M **Ensure:** a point sequence $P=\{v_{0},v_{1},\ldots ,v_{m}\}$ forming a path.   **Initialize**
1:Set current point *q* to the leftmost point in the graph**k-hop search**2:For current point *q*, among its k-hop neighbors, find a point *r* that is the closest to *q* along the time scale (the leftmost among all k-hop neighbors). If there is no k-hop neighbor, decrease *k* by 1, GOTO k-hop search. If *q* does not have any neighbors, GOTO Finish.3:Else, add point *r* into the *P* then P=P∪{r}, set current point *q* as *r*, P={q}**Finish** 4:The algorithm ends.


## 5. Optimizing the Number of Mobile Sinks and Their Paths

It is not surprising that one mobile sink cannot collect all the generated data in time if the distance between UGVs or the speed of the mobile sink is not ignored. Since the UGV will decide the meeting time according to its cruising and appearance schedule, it is useless whether or not the mobile sink arrives early or late. Once the appearance duration between two UGVs is less than their distance, there is no chance to reach them both. In this section, we try to reduce the number of mobile sinks but still achieve the same performance.

**Figure 4:**
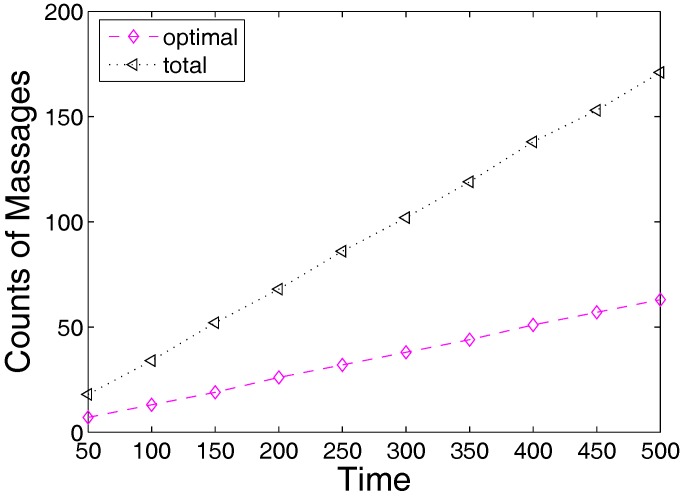
Collected data during a period.

As seen in Figure 4, even the data collection achieved by the optimal algorithm is still far from collecting all generated data. In the example, the optimal algorithm only achieves less than 1/3 of the total generated data, no matter whether the cycle is fixed or subject to the Gaussian distribution. According to the observation, we know that additional mobile sinks must be added. We bring forward some theorems before discussing the algorithm.

**Lemma** **1.** Each path is independent and has no mutual points with other paths when the number of mobile sinks is optimal.

**Figure 5:**
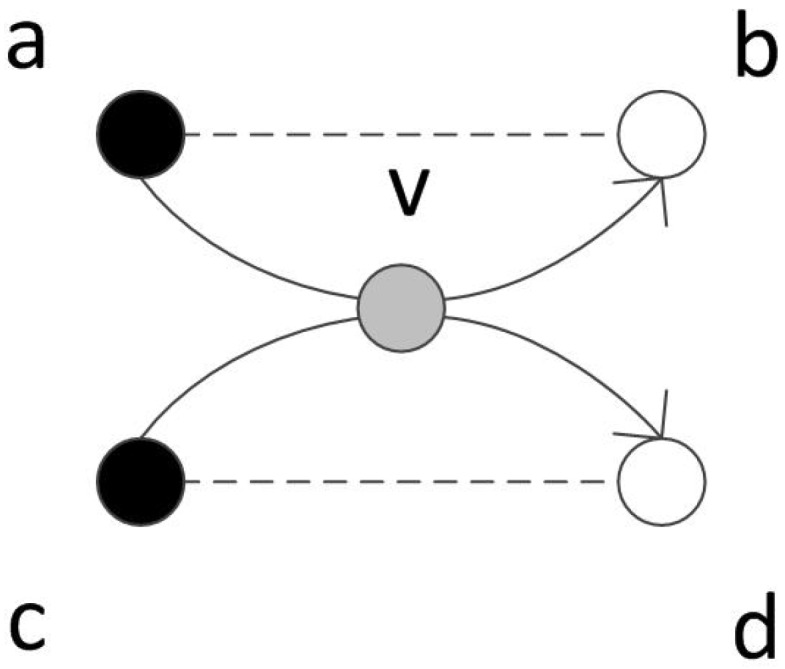
Two paths cross the same mutual point.

**Proof.**  If there are mutual points between any paths, we could summarize all the cases into the graph as shown in Figure 5 (note that *a* and *c* could be the same point just as *b* and *d* could be the same point). Two paths (*a*-*v*-*b* and *c*-*v*-*d*) pass the same point *v*, According to the triangle inequality, $d_{av}+d_{vb}>d_{ab}$ so that $t_{b}-t_{a}>d_{av}+d_{vb}>d_{ab}$, then there must be an edge between *a* and *b* which is also true for *c* and *d*. Therefore, if one path already passes the point *v*, another path could take the direct edge between ab or cd. ☐

**Lemma** **2.** Any points that have the same hop number do not have an edge between them.

**Proof.**  According to Algorithm 2, if two points *q* and *r* have an edge between them and *r* is *q*’s neighbor, then we have $s_{q}-s_{r}\geq 1$. Therefore, two hop numbers cannot be equal. ☐

**Definition** **6.** The term **connected point** means that a point v in the path has both edges on its left and its right while **unconnected point** means points with only one edge or no edges.

### 5.1. The Ideal Case

**Figure 6:**
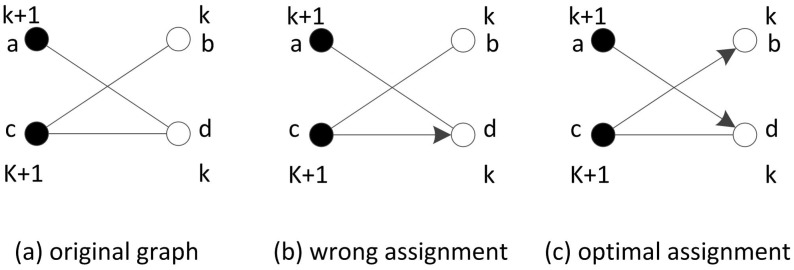
Optimal path finding via bipartite matching between points groups.

In the ideal case, as we already have the optimal path finding algorithm in the precedence graph, we will then have a graph with each point marked with a hop number. According to Lemmas 1 and 2, we know that each point will be included in the path only once and points with the same hop number do not have an edge between them. On the other hand, minimizing the number of paths equals to reducing points with a single edge or no edge (maximizing the number of connected points). Thus, we can find the optimal number of mobile sinks. We turn the problem into a maximal weight assignment in the bipartite matching where each edge has an equal weight of 1. Points of the same hop number are grouped together and adjacent hop number groups are listed on the left and right of the bipartite graph. As seen in Figure 6, the *k*th and (k+1)th hop number groups are considered, and the Kuhn–Munkras (K–M) algorithm [26] is applied to find the optimal assignment. Some points on the right may remain unsigned in the current round, and then they will be added to the next round for further consideration. Based on this, we propose Algorithm 4, which starts from the groups with hop numbers 1 and 2, then continues to 2 and 3, and so on. After all points are assigned, the algorithm ends with optimal paths. Note that there may exist more than one matching assignment that achieves the maximal total weight for each pair of hop number groups. Therefore, in the optimal case, the path distribution may be different, but the total number of paths are the same.
**Algorithm 4** Optimal number of mobile sinks in the ideal case**Require:** points with hop number **Ensure:** optimal number of mobile sinks and the paths   **Initialize** Set the weight of each edge as 1, and start from hop number 1 and 2 (*k* = 1)   **Assign**
1:If there is no point with hop number *k* + 1, the algorithm ends. Otherwise, list points with hop number *k* + 1 on the left of the bipartite graph, and those unsigned with hop number less than *k* on the right. Build the weight matrix according to all possible assignments between points derived from the precedence graph.  2:Apply the K–M algorithm, and get the optimal assignment3:Let k=k+1, repeat step 1


**Figure 7:**
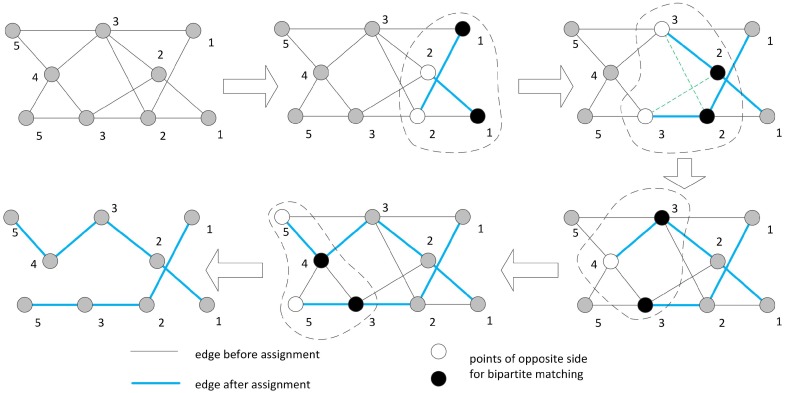
The demo shows how Algorithm 4 works.

We use the graph in Figure 3 as an example to illustrate the algorithm. The algorithm starts from the rightmost point as shown in Figure 7. First, the algorithm handles the point groups with hop number 1 and hop number 2. Then, the point groups with hop numbers 2 and 3 are listed for matching. In this step, there are two ways of assignment that have equal total weight (indicated by green dashed lines), and the algorithm randomly selects one of them. In the next step, there is only one point of hop number 4, and the algorithm also randomly selects one of two points of hop number 3 to assign with the point of hop number 4. In the final step, the unsigned point of hop number 3 joins with the point of hop number 4 to do bipartite matching with two points of hop number 5. Eventually, we get two optimal paths. It is must be noted that the optimal paths solutions are not exclusive.

**Theorem** **4.** The number derived from the Algorithm 4 is optimal.

**Proof.**  The bipartite matching between two groups implies that maximal points are linked together with edges to achieve a minimized number of unconnected points. As shown in Figure 6b, the incorrect assignment needs three vessels to visit all points (*a*-*b*, *c*, *d*) while, in Figure 6c, only two are needed (*a*-*d*, *cb*). Since the bipartite matching can achieve maximal weight [27], in each pair of point groups that is equivalent to minimal unconnected points, after all points are assigned, the algorithm can find the optimal number of paths. ☐

### 5.2. The Practice Case

In the practice case, it is not possible to develop an optimal algorithm since the duty cycle of UGVs is not constant. Even the path finding is based on the greedy algorithm. Therefore, we also propose a greedy algorithm to find the number of mobile sinks. The idea is that we also use the k-hop graph generated by the Algorithm 3 and apply Algorithm 4 on it. Since the actual appearance time of UGVs is changing, the result must be updated every time one of the mobile sinks reaches the k-hop neighbor. If there are still points remaining that are unvisited, deploy additional mobile sinks until the performance is acceptable. Algorithm 5 describes the idea.
**Algorithm 5** Finding the number of mobile sinks in the practice case**Require:** k-hop graph **Ensure:** Number of mobile sinks
1:Apply Algorithm 4 on the k-hop graph, get the paths and number of mobile sinks2:Repeat Step 1 when any mobile sink reaches the k-hop neighbor3:Add one more mobile sink if the number of unvisited points exceeds the threshold


## 6. Experimental Section

**Figure 8:**
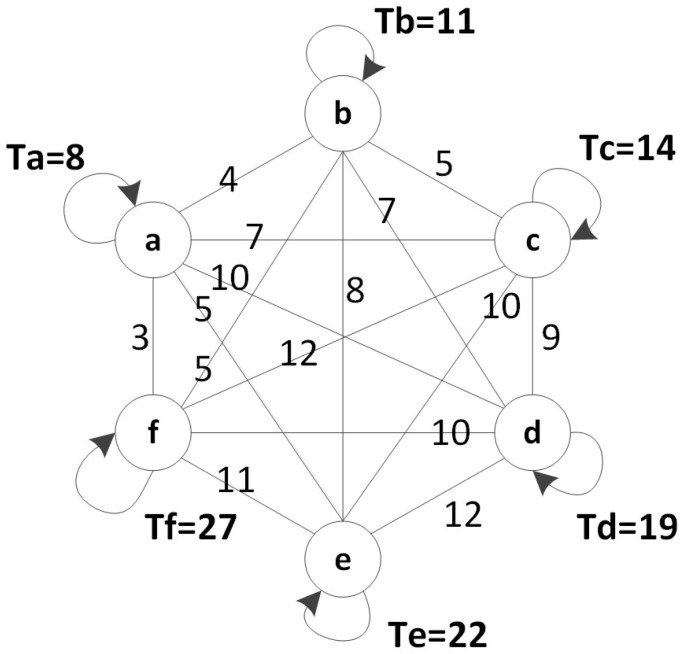
Simulation model.

In this section, we evaluate our algorithm using synthetic traces. In this trace, we repeat our experiments using a different number of UGVs which have four, six, and eight UGVs, respectively. The duty cycle of each UGV is randomly generated in Matlab. To eliminate excessive instances, we give a range as the reference of duty cycle generation. For example, in Figure 8, the range is from 5 to 30. It shows the case of six UGVs (represented by the circles) and cycles as indicated beside the circle. For simplicity, all the time are set to begin from 0. The number on the edge stands for the traveling time between surfacing locations of two UGVs. It is related to the distance and other geographical facts. In the experiments, the UGVs are randomly deployed in an x×x square, where *x* is equal to half of the upper bound of the duty cycle range. In Figure 8, the square is 15×15. All the algorithms are implemented in Matlab. We run four algorithms on this trace that are three-hop, two-hop, one-hop and random, respectively. We compare the results with the optimal solution. The cycle of each UGV abides by the Gaussian distribution. Each UGV will generate a data packet per cycle. The optimal solution in Gaussian distribution is based on the graph after each cycle is determined.

In the random algorithm, after visiting a UGV, the mobile sink will randomly choose the next destination. The one-hop algorithm will consider all neighbors of current visiting UGVs and choose the one with the lest waiting time as the next destination. Note that it is not the shortest in distance but the nearest in time for the next surfacing. The two-hop algorithm will consider the two-hop situation that will have higher costs but will bring higher efficiency. The three-hop algorithm is too costly in the practice case so it is not included in the simulation.

**Figure 9:**
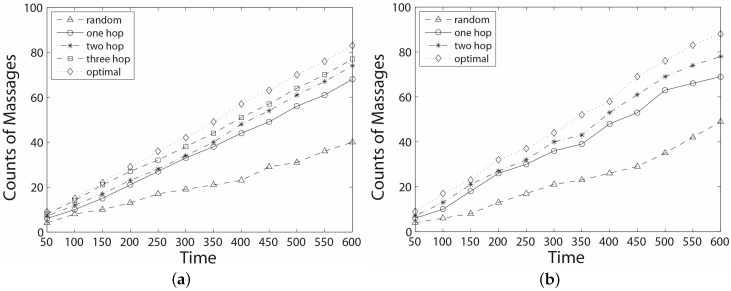
Amount of collected data (visited UGVs) during a period. (**a**) collected data in the ideal case; (**b**) collected data in the practice case.

We run the simulation along the time scale, and Figure 9 shows the collected messages vs time. In general, the random algorithm shows the lowest performance, which is only about 65% that of the two-hop algorithm. Because the one-hop algorithm has a limited view of scheduling possibility, it may choose the wrong next hop so that its performance is only better than the random algorithm. The two-hop algorithm has much better performance than the previous two. It achieves around a 7% increment compared to the one-hop algorithm. The three-hop algorithm is only 5% better than the two-hop algorithm in the ideal case. From Figure 9a, there is limited space between the three-hop algorithm and the optimal algorithm so that any algorithms that consider more hops will get very limited performance improvement but with tremendous cost. In the ideal case, since each cycle is absolutely fixed, the increment of collected data is stable. Therefore, the lines are almost linear. In the practice case as shown in Figure 9b, there are a lot of variations during the collection process and the gap between curves are bigger than that in the ideal case. However, the trend of each line is similar in both cases, and the gap is getting bigger along the time scale.

**Figure 10:**
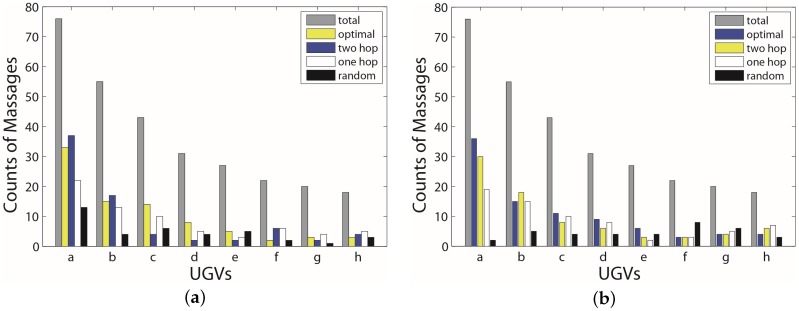
Distribution of the number of successful collected times for each UGV. (**a**) distribution in the ideal case; (**b**) distribution in the practice case.

Since each UGV has a different surfacing schedule, the one with the longer cycle will appear less times. Figure 10 shows the distribution of a number of successful collected data for each UGV (eight UGVs in total). In the figure, we could find out how many times a UGV is visited during a time period. In both figures, the random algorithm shows an unpredictable distribution. It visits *h* a lot despite the fact that *h* has the longest cycles. The one-hop algorithm has a similar pattern with that of the optimal algorithm, but it spends too much time on *h*. The two-hop has the same problem, but it gives more visits to the *a* and *b*. In terms of the optimal algorithm, UGVs with shorter cycles are visited more frequently than those that have longer cycles. From the simulation, we draw a conclusion that the more hops we consider, the better performance we will get. However, we need to keep a balance between the cost and the performance. In the practice case, the two-hop algorithm can achieve good performance compared with the cost.

**Figure 11:**
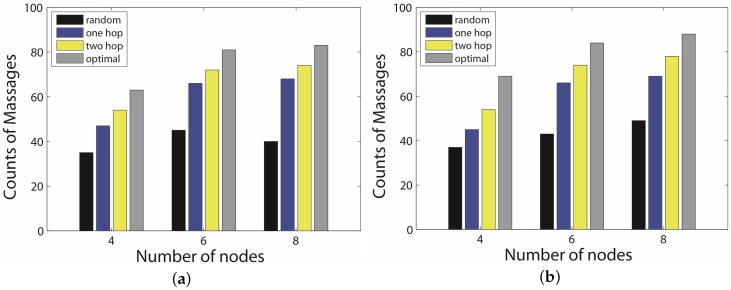
Performance comparison of different number of UGVs. (**a**) distribution in the ideal case; (**b**) distribution in the practice case.

We also did experiments on a different number of UGVs. Both figures of Figure 11 show that, with the increasing of hop counts considered, a higher performance will be achieved. In addition, in the practice case, all algorithms except the random can collect a little more data than in the ideal case, mainly because the uncertainty makes the duration more elastic. When the total number of UGVs is four, the random algorithm only has the performance of about 60% as the optimal algorithm, while the one-hop algorithm is about 78% and the two-hop is about 90% as the optimal. An interesting finding is that, with the increasing of the number of UGVs, the absolute difference of data collected from the one-hop, the two-hop and the optimal algorithms remains almost the same, which means that the larger the number, the higher performance our proposed algorithms can achieve. However, we need to keep a balance between the cost and the performance.

**Figure 12:**
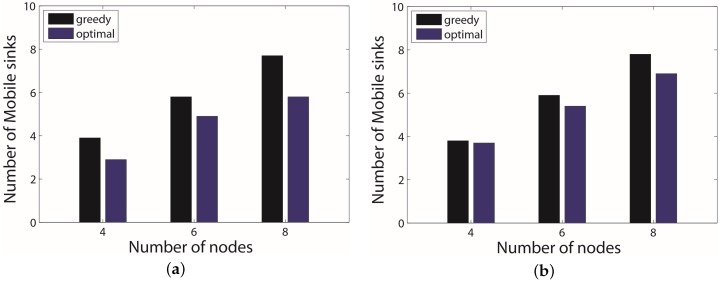
Experimental results of mobile sinks optimization in the ideal case and practice case. (**a**) number of mobile sinks in the ideal case; (**b**) number of mobile sinks in the practice case.

To evaluate the performance of mobile sinks optimization, we conducted experiments in the ideal case and the practice case. In each case, we apply the algorithm when the number of UGVs was four, six and eight, respectively. For each group of fixed number of mobile sinks, we use 10 different settings and calculate the average. We name our algorithm *optimal* and compare it with another algorithm called *greedy*, which is a strategy is try to connect the most adjacent points first during a path constructing in the graph with a marked hop number. From Figure 12, we can see that the *greedy* algorithm can hardly reduce the number of mobile sinks, since, in both cases, the numbers of mobile sinks are almost equal to the number of UGVs. The *optimal* algorithm achieves better performance in both cases. It tends to save more mobile sinks when the number of UGVs increases. However, in the practice case, since the behavior of UGVs cannot be accurately predicted, it can reduce a lower number of mobile sinks than that in the ideal case.

## 7. Conclusions

In this paper, we have studied a special application of data collection in widely distributed sensor networks. The scenario is that a mobile sink cruises on the ground to collect data gathered from Unmanned Ground Vehicles. The practice case could be a mobile sink based data collection in a duty-cycled sensor network, a hard accessible sensor network, or a surface vessel based under water data collection in the deep sea. In these networks, there is only a short time window for each sensor node appearing to meet the mobile sink. Once the window is missed, the data collection latency increases a lot. We address the unique challenge and propose two algorithms to schedule the path of the mobile sink, targeted at visiting the maximum number of UGVs in a timely manner with the shortest path, according to the timing constraints bound by the cycles of UGVs. Then, it is extended to the case that the cycle timing of UGVs cannot be precisely predicted or measured. The path decision is then made on the k-hop knowledge of the environmental parameters. Furthermore, we develop two algorithms to optimize the number of UGVs deployed in the ideal and practice cases, respectively. Simulations are conducted with a random algorithm and the deadline driven algorithm to evaluate the performance of the proposed algorithm. In some cases, more mobile sinks should be introduced to collect the data in time since even the optimal algorithm could not gather them all. In the future, we will further improve the performance of our path scheduling algorithms with the same or less order of the computational cost.
